# Implementation of an ionization chamber array for linear accelerator monthly dosimetry QA

**DOI:** 10.1002/acm2.14433

**Published:** 2024-06-23

**Authors:** Sameer Taneja, David L. Barbee

**Affiliations:** ^1^ Department of Radiation Oncology New York University Langone Medical Center New York New York USA

**Keywords:** dosimetry, IC Profiler, linear accelerator QA, monthly QA

## Abstract

**Purpose:**

The IC Profiler (ICP) manufactured by Sun Nuclear Corporation (SNC) is an ionization chamber (IC) array used for linear accelerator dosimetry measurements. Previous work characterized response of the ICP under various conditions, but there is limited work of its implementation into monthly QA measurement procedures. This work quantifies ICP accuracy and variables that affect accuracy for beam output measurements, and demonstrates feasibility of using the ICP for all recommended monthly dosimetry measurements.

**Methods:**

A total of 1985 output measurements on six Varian TrueBeam and Edge linear accelerators were performed using three ICP with quad wedges (QWs) and were compared with conventional IC measurements. The accuracy of the ICP for beam output was characterized as the difference between the ICP and IC. Variables that affect ICP accuracy, including gain settings, calibrations, and template baselining as well as machine or energy‐specific bias were investigated. Measurements of profile constancy, energy, dose rate constancy, wedge factors, and gating were performed.

**Results:**

The initially observed mean output difference between the ICP and IC was 0.16% (0.61%). When gain settings were optimized, the output difference accuracy improved to −0.02% (0.38%). The output accuracy of the ICP was not dependent on array, dose, temperature and pressure calibrations, or template baselining. Statistically, ICP output accuracy was dependent on machine and beam energy, but clinically, all measurements fell within 0.5% of unity. ICP measurements of energy, dose rate constancy, and wedge factors matched passing results with conventional IC in water measurements. Gating and beam profile constancy measurements demonstrated good stability using the ICP. Finally, monthly dosimetry QA using ICP was completed in an average of 33 min compared to 66 min using the IC.

**Conclusion:**

This work demonstrated the feasibility and efficiency of using the ICP, with specific considerations, as a measurement device for dosimetric linear accelerator monthly QA.

## 1 INTRODUCTION

A comprehensive linear accelerator quality assurance (QA) program confirms that machine parameters have not deviated outside of tolerance from the time of acceptance and commissioning,^1^, ^2^, ^3^, ^4^ thereby ensuring treatment accuracy during delivery of a variety of patient therapies. Currently, guidelines for specific QA tests for individual components of a linear accelerator are given by the American Association of Physicist in Medicine (AAPM) Task Group (TG)‐142^3^ with an implementation guide provided by AAPM TG‐198.^4^ According to tab. 2 in the AAPM TG‐142 report,^3^ monthly QA dosimetry tests performed by a qualified medical physicist (QMP) include photon and electron output constancy, beam energy constancy, dose rate output constancy, and beam profile constancy, with additional wedge factor and gated output constancy checks as needed. Conventionally, these dosimetric measurements are completed using a Farmer‐type ionization chamber (IC) and electrometer either under AAPM TG‐51^5^ conditions or through a non‐water solid phantom or jig using a factor that has been cross‐calibrated to a reference dosimetry measurement traced to an accredited dosimetry calibration laboratory (ADCL) system. Performing monthly QA measurements using these systems can be time‐consuming and labor‐intensive, whereby a one‐to‐one relationship exists between each measurement and a single QA task^6^ with certain tasks requiring modification of measurement conditions and repeated trips into the treatment vault for setup modifications.

Risk based analysis and prioritization of tests have been applied to linear accelerator machine QA.^7^ Smith et al.^8^ used a risk analysis method to highlight linear accelerator QA tests that are most effective at maintaining safety and quality of patient care. Risk priority number (RPN) scores showed that the highest severity for QA tests outlined in TG‐142 was for machine output constancy for both daily and monthly tests, indicating the need for accurate output constancy measurements.

This work focuses on the use of an IC array phantom, the IC Profiler (ICP) manufactured by Sun Nuclear Corporation (SNC, Melbourne, FL), for measurement of monthly QA‐required beam parameters. Simon et al. provided^9^ an in‐depth description of the ICP along with an initial characterization of the device. For linear accelerator measurements, the ICP showed good short and long‐term reproducibility, minimal dose dependence greater than 20 MUs, minimal dose rate dependence above 8 cGy/s, and minimal backscatter contribution.

The ICP has been in clinical use for well over a decade, during which time its scope and utilization has continuously expanded. Multiple work from Gao et al.^10^, ^11^ and Goodall et al.^12^ used the ICP to measure beam energy for photons and found that metrics determined from measured photon field profiles along the diagonal axis using an ICP were more sensitive to energy changes than central axis energy measurements with an IC at multiple depths. In additional work, Gao et al.^13^ used the ICP for electron beam energy determination using dual aluminum energy wedges along the positive diagonal to determine the *R*_50_ by deriving a linear relationship between *R*_50_ and the normalized full‐width half maximum (FWHM) of the attenuated axes. They reported a 3‐year experience showing ICP measured *R*_50_ matched expected *R*_50_ within 0.1 cm and noted that flatness and symmetry were unaffected by the presence of dual wedges.

SNC later expanded on the wedge concept for the ICP and introduced quad wedges (QWs) which provide wedge attenuation on both positive and negative diagonal axes. The ICP with QWs system was used to determine an AreaRatio variable, calculated as the relationship of the area under the attenuated profiles normalized by the unattenuated profile area, and can be correlated to beam quality metrics *D*_10_ and *R*_50_ for photons and electrons, respectively.^14^ Gao et al. confirmed the accuracy of *D*_10_ calculations using the ICP with QWs for five Varian Halcyon machines using a 6 FFF energy in an inter‐institutional study.^15^

In addition to beam output constancy and beam energy, beam profile constancy can be measured using the ICP. Simon et al.^9^ showed that beam profile measurements matched to approximately 0.75% compared with an IC in water. Gao et al.^16^ compared symmetry of beam profiles using the ICP and water tank measurements and found that 95% of point‐by‐point symmetry comparisons agreed within 0.7%.

There are many efforts to automate monthly QA beam output measurements using non‐water tank measurements using EPIDs, phantoms, or device‐based measurements. Skinner et al.^6^ implemented the ICP with QWs into monthly QA procedures, and compared beam output measurements with water tank measurements in the standalone SNC Profiler software to water tank measurements over a time period of 6 months and across four machines. For beam output, the difference between ICP and IC outputs had a standard deviation in percentage difference between measurement techniques of 0.3% for photons and 0.3%–0.5% for electrons.

This work presents a 4‐year institutional experience with implementation of the ICP for all monthly dosimetry QA for an institution with multiple users and linear accelerators using a novel vendor software QA platform that directly interfaces with ICP. ICP measurements of beam output, in dose/MU, were compared with reference IC measurements to determine the accuracy of the ICP. Factors affecting ICP accuracy in the institution's initial implementation of the ICP are discussed, including most notably the variability in gain setting between each energy measurement. The ICP and IC comparison was further investigated to determine whether beam output accuracy demonstrated dependency on machine or electron or photon energy. Additional monthly dosimetric QA measurement stability was assessed for beam energy, beam profile constancy, dose rate constancy, wedge factor, and gated constancy and compared to IC measurements and expected profile constancy. Lastly, the QA tracking software was used to track the time required to perform ICP and IC measurements, separately, to quantify the time spared by using the ICP for monthly QA dosimetry tasks.

## 2 METHODS

Monthly QA dosimetry was performed on six linear accelerators spanning three clinical sites between December 2018 and August 2022. All measurements were completed by eight QMPs^8^ that rotated machine coverage each month. Linear accelerators included five TrueBeam and one Edge, manufactured by Varian Medical Systems (Palo Alto, CA). All machines were equipped with four photon energies (6 MV, 6 FFF, 10 FFF, and 15 MV), two machines were equipped with an additional 10 MV photon energy, and four machines were equipped with electrons (6, 9, 12, 16, and 20 MeV).

### 2.1 Output constancy measurements

Linear accelerator monthly output measurements were conducted using two primary devices: an ADCL calibrated IC and the ICP with QWs. All measurements were performed using SunCHECK Machine (SNC) using templates for monthly dosimetry generated with tasks designed for QA tasks with an IC and with integrated ICP with QWs. Measurements using the two devices are described:

#### 2.1.1 Farmer type ionization chamber

The first method of dosimetry QA measurements involved placing a Farmer Type IC, either an Exradin A12 (Standard Imaging Inc., Middleton, WI) or a PTW 30013 (PTW, Freiburg, Germany), calibrated by an ADCL, in a 30 × 30 × 30 cm^3^ 1DS water tank and PC Electrometer (SNC) system. 100 MU were delivered with the IC set to TG‐51 reference conditions with a chamber placed at 10 cm depth, 100 cm source‐to‐surface distance (SSD), and a 10 × 10 cm^2^ field size for photons and *d*_ref_ depth, 10 cm SSD, and a 10 cm cone for electrons. A calculation of output in dose/MU [cGy/MU] was calculated in SunCHECK Machine (SNC) using an ion chamber reading, *N*_D,w_ coefficient, pressure and temperature corrections, and TG‐51 factors,^5^, ^17^ shown in Equation (1):

(1) $$\;\frac{\mathrm{Dose}}{\mathrm{MU}}=\frac{\mathrm{RDG}*N_{D,w}*\mathrm{PTP}*\mathrm{TG}-51}{\mathrm{MU}}.$$

In Equation (1), the TG‐51 factors are comprised of *P*_pol_, *P*_ion_, *k*_Q_, and PDD (%) for photons and *P*_pol_, *P*_ion_, *P*_gr_, *K_R_*_50_, *K*_ecal_ and PDD (%) for electrons.^5^, ^17^ These factors were measured during annual QA or after an IC repair. The corresponding SunCHECK Machine monthly ion chamber templates were updated to include all chamber TG‐51 parameters and factors into a single template.

#### 2.1.2 IC Profiler with quad wedges

**FIGURE 1:**
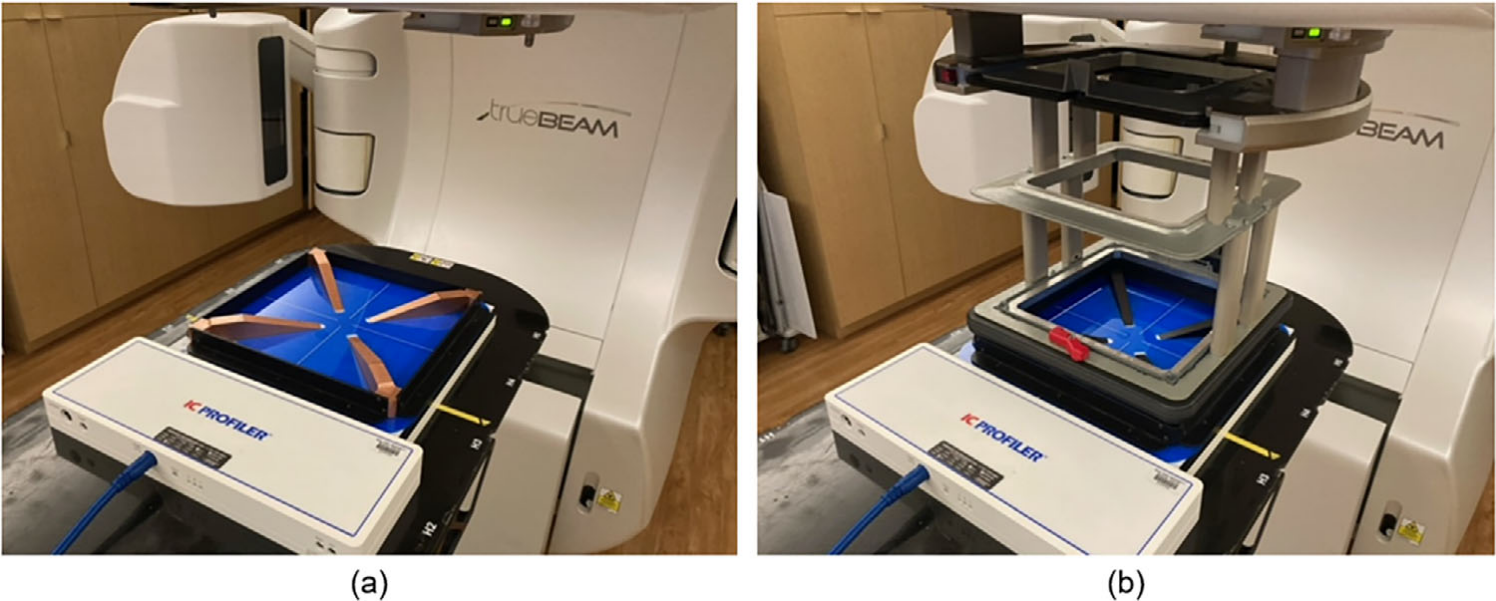
IC Profiler with quad wedge attachment orientation and setup for measurements for (a) photons and (b) electrons.

The second method of beam constancy measurement was completed using the ICP with QWs. In this study, all ICP measurements were performed with QWs placed directly on the ICP (Figure 1). QWs consist of four metal wedges fixed diagonally in a square, aluminum frame and mounted on an acrylic plate with 0.3 cm thickness such that the edge of the plates were placed flush with the face of the ICP. The wedges reduce in thickness from the outer edges to the center having no material present in the center of the wedge to avoid affecting central axis dosimetry measurements. The wedge material was made of copper and aluminum for photon and electron measurements, respectively. The ICP was setup on the linear accelerator couch with the light field cross hairs on the ICP principle axes and an SSD of 100 cm. The QW for the chosen modality was placed flush to the ICP surface, centered to the central axis with no additional buildup or backscatter material was used. This configuration was irradiated using 100 MU and with field sizes recommended by SNC for calculating the AreaRatio variable for beam energy: 30 × 30 cm^2^ for photons and a 25 cm cone for electrons. The treatment couch and ICP were not moved when installing the electron applicator or changing QWs when switching radiation modalities.

Prior to establishing baselines in SunCHECK Machine, each ICP used in this work established three calibrations for array, dose, and temperature and pressure as per manufacturer recommendations. Array calibrations were performed for each energy in a standalone Profiler software and uploaded to SunCHECK Machine. Dose and temperature and pressure calibrations were performed directly in SunCHECK Machine. Once initial calibrations were completed, all three calibrations were stored in a combination of SQL and Mongo databases that linked the calibrations to the ICP serial number. Template baselines in SunCHECK Machine were initially established for each machine and energy by delivering a single 100 MU irradiation to the ICP. Warning and failure tolerance levels were subsequently set to each QA task following recommendations from TG‐142.

Routine ICP and QW measurement consisted of setting up the device for irradiation and creating a new instance from the monthly QA template in SunCHECK Machine. When the ICP is connected, the template recognizes the ICP prompting a 30 s background collection for leakage correction. The background, along with specific calibrations and QA task baseline and tolerances are updated and applied to a given measurement.

#### 2.1.3 Measurement dataset

A total of 1985 paired ion chamber and ICP measurements were performed using SunCHECK Machine for all energies and machines over a time period ranging from December 2018 to August 2022. All monthly QA ICP and IC measurements were retrieved directly from SunCHECK Machine's SQL database through custom queries and verified in the SunCHECK web‐based frontend. Three months of data (March 2022–May 2022) for three machines were excluded from the dataset as the ICP measurements were not completed when a center's ICP was under repair. For months that required an output adjustment, the ICP measurements were completed after beam output adjustment and were compared with the postadjustment ion chamber values.

The ICP beam output accuracy variable referred to throughout this work was determined by comparing the results of the ICP measurement and the IC measurement, expressed as dose/MU, and both measurements were completed during the same monthly QA. The ICP accuracy was described as a difference in percentage between the ICP and IC measurements, and is presented in Equation (2).

(2) $$\mathrm{Output}\_\mathrm{Dif}\left(\%\right)=\left(\mathrm{ICP}-\mathrm{IC}\right)*100\%.$$

The Output_Dif calculated for all measurements is referred to throughout this work as ICP accuracy. ICP calibrations for temperature and pressure, array, and dose were completed using the manufacturer's recommendations prior to the ICPs implementation into monthly QA procedures and after any ICP repair. All three of these calibrations were completed together and were completed twice during the course of this study for each ICP. A Student's two‐tailed *t*‐test was used to determine the significance of changes in ICP accuracy pre‐ and postcalibrations.

The ICP template in SunCHECK Machine was re‐baselined on a periodic basis: prior to ICP introduction into monthly QA procedures, post any ICP repair or hardware change, and on an annual basis after linear accelerator annual QA, in which both beam steering and TG‐51 were completed. A Student's two‐tailed *t*‐test was used to evaluate the significance of changes in ICP accuracy due to template re‐baselining.

### 2.2 Gain settings

During the time period of this study, there was one adjustment made to the clinical workflow that affected the accuracy of the ICP output measurement. Initial implementation of the ICP with SunCHECK Machine were set using variable gain settings per energy in order to maximize signal from variable dose rates. Photon output measurements were initially performed with gains of 4, 2, 4, 1, 4 for energies of 6 MV, 6 FFF, 10 MV, 10 FFF, and 15 MV, respectively, based on the maximum achievable dose rate and measurement dose rates of 600 MU/min for flattened beams, 1400 MU/min for 6 FFF, and 2400 MU/min for 10 FFF. All electron energies used a gain of four corresponding to their measurement dose rate of 1000 MU/min.

The gain is set in SunCHECK Machine and is used as a multiplier by the raw counts measured by the detector. That multiplier, in addition to the lapsed time since starting the measurement, the leakage rate, the relative array calibration, and the dose per count are used in the calculation of the absolute dose for a detector. During initial implementation of SunCHECK Machine as the driver for ICP measurements, gain settings were set as described above to match the gain settings used in the Profiler standalone application. Unlike the standalone software, SunCHECK Machine did not prompt the user to perform a new background measurement when changing gain settings. As the gain impacted the background correction and ultimately the measurement, this led to inconsistent factors being applied across measurements. All templates were re‐baselined with a uniform gain of 1 for all photons and electrons. The difference between ICP and IC measurements (Equation 2) were analyzed pre‐ and postgain adjustments to determine impacts on ICP accuracy

### 2.3 Machine and energy dependence

The ICP was clinically implemented across three campuses that utilize six different linear accelerators with varying energies. In order to determine if ICP accuracy was consistent across all machines and energies and that a single variable was not skewing the overall dataset, two‐way ANOVA tests were performed to evaluate the difference in accuracy of the ICP across machines and energies. Although the gain would not be expected to impact machine and energy dependence, these tests were performed for the two datasets described in the previous section (non‐uniform gain and uniform gain of 1) as they are representative of the full ICP dataset with variable gains and the adjusted, more accurate single gain dataset. For a more clinically relevant comparison, the ICP accuracy values were also parsed based on machine and energy.

### 2.4 Monthly QA measurement procedures

To fulfill TG‐142 dosimetry QA recommendations for monthly QA, four 100 MU measurements were needed to measure all photon parameters for each energy: (1) beam output, beam energy, beam profile constancy, (2) second dose rate constancy, (3) wedge factors, and (4) gating constancy. Electron dosimetry required a single 100 MU measurements using a dose rate of 1000 MU/min, which acquired beam output, beam energy, and beam profile constancy. Beam profile constancy and energy were baselined in the same measurement as beam output. An additional 100 MU irradiation at the machine's largest field size of 20 × 20 cm^2^ with a 60° dynamic wedge was needed to calculate the wedge factor for all flattened photon beams. The wedge factor was normalized in SunCHECK Machine to the open 30 × 30 cm^2^ field measurement. As the field sizes were mismatched between the wedged field and the open field, output factors for both field sizes and percent depth dose curves from machine commissioning measurements were used to confirm the ICP wedge factor with the ionization wedge factor prior to clinical implementation. Subsequent ICP measurements used the baseline value. During the course of the study, the baseline for the wedge factor was unchanged and all machines were set to the same wedge factor baseline for each energy. Dose rate and gating constancy were compared relative to beam output and did not require an additional baseline.

Beam profile constancy was characterized by symmetry and flatness values in the x‐ and y‐axis, calculated in 80% of the field size. Symmetry was calculated as a normalized point difference and flatness was calculated using the ratio of the maximum and minimum values in the flat region of the field size, with both parameters expressed in percentage. Profile constancy measurements were introduced in 2020 during an upgrade of SunCHECK Machine, and set as part of monthly QA protocols late 2020 to early 2021 for a total of 1026 measurements. Machine wedge factors were measured for enhanced dynamic wedges (Varian Medical Systems) for the steepest wedge angle of 60°. Beam energy was described using *D*_10_ [%] for photons and *R*_50_ [cm] for electrons and was measured using the copper and aluminum QWs, respectively.

Profile flatness and symmetry, wedge factors, and beam energy measured using the ICP were compared with baseline values that were set postbeam steering, output adjustment, and beam measurements, respectively, which occurred on an annual basis when the template was re‐baselined. The IC was not used monthly for beam profile constancy measurements.

Dose rate constancy was measured for a secondary dose rate for all photon energies. The secondary dose rate for photon energies with flattening filters (6, 10, and 15 MV) was 300 MU/min, and for 6 FFF and 10 FFF was 800 MU/min and 1200 MU/min, respectively. No secondary dose rate for electrons was measured. The dose constancy QA test in this work is presented as a percent difference between primary and secondary dose rates. A total of 1275 dose rate measurements were analyzed.

The last test that was measured by the ICP to complete all required TG‐142 dosimetry monthly QA was gating constancy. This measurement was only completed using the ICP and not with the IC as introduction of gating QA with ICP coincided with institutional implementation of treatments using breath hold. Gating constancy measurements were completed by setting up a phantom with multiple reflector BBs used for real‐time position (RPM, Varian Medical Systems) on a phantom that mimicked a constant breathing motion. A QA plan was developed with gating enabled, and was run for each energy using respiratory phase gating with simultaneous ICP measurement. The gating constancy was compared with the beam output value and is presented as percent difference. The implementation of gating QA using the ICP into institutional procedures occurred in late 2020 and was performed specific linear accelerators based on whether the machine was used for gating procedures. As a result, a total of 930 measurements across all photon energies were analyzed in this work.

Conventional ion chamber in water measurements were performed using the same measurement setup as reference output. Beam energy was assessed at two depths, *D*_20_/*D*_10_ for photons [%] and at *R*_50_ for electrons normalized by charge at *d*_ref_ [%] with both compared to expected clinical ratios based on commissioning data. Dose rate output was measured at the same reference conditions with the same dose rates used in ICP measurement. Wedge factors were measured using 60° EDW and 10 × 10 cm^2^ field size at a depth of 10 cm and compared to commissioning data. Gating output was not measured using ion chamber in water.

### 2.5 Time analysis

The time required to perform complete beam output constancy measurements was tracked individually for both techniques using the ICP and the IC in a water tank using timestamps in SunCHECK Machine. As multiple beam parameters were determined with a single ICP measurement, the ICP parameters measured during these timestamps included beam constancy, energy, profile constancy, wedge factor, and secondary dose rate constancy. The ion chamber in water measured the same parameters with the exception of profile constancy. Note that gated constancy was not included in these measurements, as it was not completed for the IC in water setup.

The timestamp dataset was queried from the SunCHECK Machine SQL database. The database tracks the starting time for the first measurement, and only included data from the first to last penultimate of the executed template. As there were five electron energies used and four photon energies used and timestamps were available for three photon energies and four electron energies, the total amount of time was corrected. The overall time was adjusted to account for that by a scaling factor of 1.33 for photons and 1.25 or electrons. The resulting time intervals were validated against timestamps in the SunCHECK Machine platform and through individual timed testing.

All months were included in the analysis except for months in which either ICP or ion chamber measurements took >45 min, indicating there was an issue with the ICP or machine and for months that required linear accelerator output adjustment as that increased the IC measurement time. A total of 66 months were included in the dataset. Mean and standard deviations of the time to complete the first measurements were reported separately for photons and electrons. The setup and breakdown time allotments were estimated.

## 3 RESULTS

### 3.1 Output constancy accuracy

**FIGURE 2:**
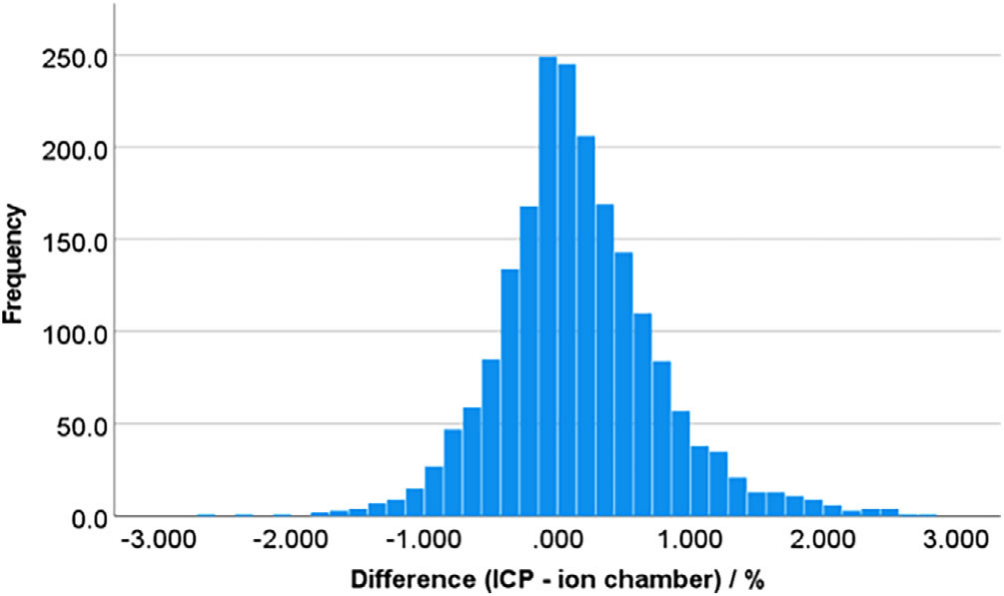
Histogram showing the difference between IC Profiler (ICP) and ion chamber measurements for the full dataset (*N* = 1985). The mean difference was 0.16% with a standard deviation of 0.61%.

The accuracy of the ICP for beam output, characterized by the difference between the ICP measurement and the IC (Equation 2), is shown in a histogram in Figure 2 for all machines and all energies in the entire 4‐year dataset (*N* = 1985). The mean deviation and standard deviation was 0.16% and 0.61%, respectively. Nineteen measurements (0.9%) showed a difference between ICP and ion chamber measurements of greater than 2% and 63 measurements (3.2%) were greater than 1.5%. Institutional tolerances involve adjusting the beam output when ion chamber measurements are greater than 2%, as per TG‐142 recommendations. The ICP measured six instances greater than 2% while IC measurements were less than 2%, indicating a false positive output adjustment rate of 0.3%. All of these measurements were FFF energies and all instances were measured prior to standardizing the ICP measurement gain to one for all energies. Similarly, three instances, or 0.2% of the cases, showed a false negative output adjustment, which was defined as the IC reading above the 2.0% threshold while the ICP did not. Similarly, all three cases were measured prior to standardizing the ICP measurement gain to one. If the beam adjustment threshold was set to 1.5% from unity, the false positive rate was 2.0% and the false negative rate was 0.2%.

The impacts of ICP calibrations for array, dose, and temperature/pressure on ICP accuracy was investigated. All three calibrations for the ICP were completed prior to implementation of the ICP and data collection in 2018 and during the month of February 2021, forming two datasets of ICP accuracy to compare. The mean difference and standard deviation prior to February 2021 and post February 2021 was 0.11% ± 0.52% and 0.06% ± 0.47% for all combined energies and machines. A Student's *t*‐test demonstrated that the difference in means between these distributions was not statistically significant (*p* = 0.15). These results indicated ICP accuracy is independent of ICP calibrations. Note that calibrations of array, dose, or temperature and pressure were not completed separately. Similarly, the ICP template was baselined and re‐baselined during annual QA months. A univariate analysis of variance was performed and found no statistical deviation pre–post template baselining (*p* = 0.18).

### 3.2 Gain settings

**FIGURE 3:**
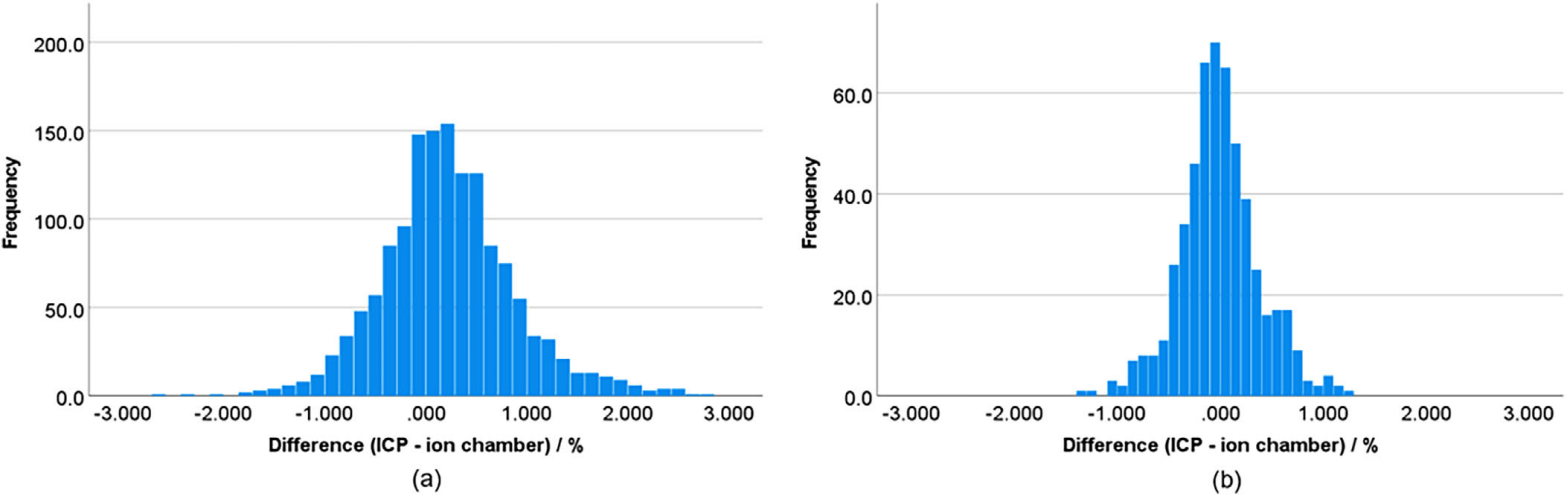
Histograms showing the difference in percentage between IC Profiler (ICP) and ion chamber measurements for (a) variable gain settings based on dose rate (*N* = 1452) with a mean of 0.22% and a standard deviation of 0.66% and (b) with the gain set to 1 for all energies (*N* = 533) with a mean of −0.02% and a standard deviation of 0.38%.

Figure 3 shows a histogram of the ICP and IC accuracy for the full dataset presented in the previous section but separated for measurements completed with (a) the institutions initial setup of using different gains based on beam energy (*N* = 1452) and (b) after the gain was changed to one for all energies (*N* = 533). The mean difference was reduced from 0.22% to −0.02% and the standard deviation was reduced from 0.66% to 0.38%. Postgain adjustment, no differences between ICP and IC greater than 1.5% were observed. Additionally, there were no false positives and false negatives in this data set, indicating no failure in identifying needed adjustment or incorrectly prompting adjustment, respectively. A two‐tailed unpaired Student's *t*‐test was used to test the hypothesis that the mean value of the ICP accuracy was different between data collected with variable gain and for a gain set to one for all energies. Difference was considered significant if *p* < 0.05. The result of the Student's *t*‐test showed a mean difference between the ICP accuracy between the two groups (*p* < 0.001).

Similar Student's *t*‐tests were run to investigate the effects of the gain setting during measurement on all other dosimetric parameters measured during monthly QA with the ICP. These included energy constancy for photons and electrons, flatness, symmetry, dose rate, wedge factor, and gating constancy. There was no significant results with the exception of x‐ and y‐symmetry, which indicated that the gain setting significantly changed the measurements of symmetry. The mean x‐ and y‐symmetry (standard deviations) were 0.03% (0.23%) and 0.10% (0.31%) pregain setting (*N* = 493) and 0.06% (0.21%) and 0.06% (0.18%) postgain adjustment (*N* = 533), respectively. In addition, once the gain was set uniformly to zero, the number of symmetry constancy values that were greater than TG‐142‐recommended 1% tolerance was reduced from 14 to 0.

### 3.3 Machine and energy dependence

Two‐way ANOVA tests were performed in statistical package for the social sciences (SPSS, IBM, Armonk, NY) to test if the accuracy of the ICP were machine‐ and/or energy‐dependent. The two‐way ANOVA evaluated the hypothesis that the ICP accuracy (Equation 2) varied with the machine and energy used for measurement. Statistical significance was taken as having *p* < 0.05. The test also assessed interaction between machine and energy terms, and when statistically significant, this interaction term indicated that the expected ICP accuracy is dependent on both machine and energy. Test statistics, degrees of freedom, and individual *p*‐values are reported in Table 1. The two‐way ANOVA showed that the difference between ICP and IC results varied with both machine and energy (*p* < 0.001). While these two‐way ANOVA results were performed using the dataset postswitching the gain to 1 (Section 3.2) as it is representative of ICP accuracy (*N* = 533), they were consistent with the overall dataset (*N* = 1985).

**TABLE 1:** Detailed statistical results for two‐way ANOVA test used to evaluate if the ICP and IC difference are machine or energy dependent.

	Machine			Energy			Interaction		
Test	*F*	*df*	*p*	*F*	*df*	*p*	*F*	*df*	*p*
Difference (gain 1, *N* = 533)	35.35	5	<0.001	4.57	9	<0.001	2.60	31	<0.001
Difference (variable gain, *N* = 1985)	17.06	5	<0.001	5.23	9	<0.001	1.61	31	0.018

For each main effect (machine, energy) and for the interaction term between the two factors the following are reported: Test statistic (*F*), degrees of freedom (*df*), and *p*‐value (*p*). ANOVA, analysis of variance; IC, ionization chamber; ICP, IC Profiler.

**FIGURE 4:**
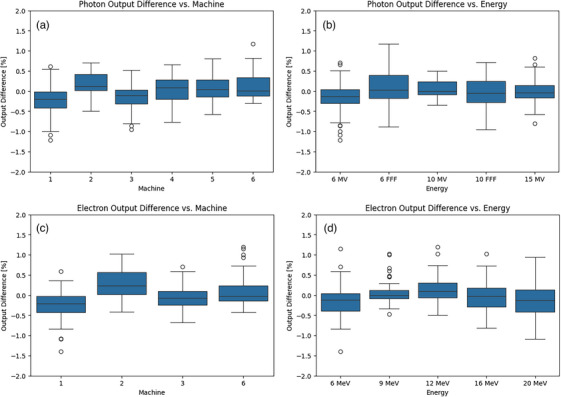
Boxplots showing the difference in output between the IC Profiler (ICP) and ionization chamber (IC) for the gain = 1 dataset, parsed for machine and energy for (a, b) photons and (c, d) electrons.

Figure 4 shows boxplots representing the calculated difference between ICP and IC measurements for each energy, photons and electrons, and machine, respectively. The mean difference between ICP and IC for machines 1−6 averaged for all energies (photons and electrons included) was −0.24%, 0.25%, −0.10%, 0.03%, 0.10%, and 0.11%, respectively, and standard deviations ranged from 0.29% to 0.37%. For photon energies, the mean difference for 6 MV, 6 FFF, 10 MV, 10 FFF, and 15 MV were −0.15%, 0.05%, 0.08%, −0.02%, and −0.01%, respectively. Similarly for electron energies, the mean difference for 6, 9, 12, 16, and 20 MeV were −0.13%, 0.07%, 0.15%, −0.01%, and −0.13%, respectively.

A similar two‐way ANOVA analysis was completed for all monthly QA dosimetric parameters measured with the ICP. Dose rate, wedge factor, and gating measurements did not show significance with either machine or energy. Photon and electron showed statistical differences with machine but not energy or the interaction variable, and beam profile constancy parameters showed statistical differences with both energy and machine. Although significance was seen, breakdown of machine and energy values are presented to evaluate clinical variation.

### 3.4 Additional dosimetry monthly QA measurements

Beam profile constancy, defined by x‐ and y‐axes flatness and symmetry, was defined as the measured parameter subtracted by the baseline, in percent and was measured using the ICP. The symmetry parameter was affected by the gain setting as described in Section 3.2, the beam profile constancy was presented for ICP measurements with the gain uniformly set to 1 (*N* = 533). For x‐flatness, y‐flatness, x‐symmetry, and y‐symmetry, the mean deviation (standard deviation) was −0.00% (0.15%), 0.04% (0.15%), 0.06% (0.21%), and 0.06% (0.18%), respectively. The maximum deviation of the mean value for flatness and symmetry (in both x‐ and y‐directions) parameters was 0.07% for flatness and 0.3% for symmetry across all machines and 0.1% for flatness and 0.1% for symmetry across all energies.

Beam energy constancy was defined as the measured *D*_10_ or *R*_50_ subtracted by the baseline, as percentage and depth, respectively. ICP measurements across all machines and energies showed a mean *D*_10_ constancy of −0.02% with a standard deviation of 0.30% (*N* = 1113) and a mean *R*_50_ constancy of 0.01% with a standard deviation of 0.05% (*N* = 872).The maximum deviation in the mean photon and energy constancy between machines was 0.1% and 0.02 cm and between energy was 0.02% and 0.02 cm, respectively.

Secondary dose rate constancy or all photon energies were measured using the ICP. The mean reading was 0.3% (*N* = 1275), with no failures set to a tolerance of ±2%. In addition, the average wedge factor value (baseline value) was 0.411 (0.41) for 6 MV (*N* = 256), 0.461 (0.46) for 10 MV (*N* = 46), and 0.481 (0.48) for 15 MV (*N* = 257). There were no wedge factor measurements outside the tolerance of ±2%. Dose rate and wedge factor results were consistent with IC measurements. Finally, gating constancy measurements were compared with beam output measurements, both using the ICP, and presented as a percentage difference. Across all photons, the mean percentage difference was 0.02% with a standard deviation of 0.36%. No IC measurements for gating constancy were performed during this time.

### 3.5 Measurement time

Table 2 shows the measurement efficiency of the ICP when compared with IC in water measurements. Overall time was broken down into device setup, measurement time, and breakdown. Timestamps from SunCHECK Machine showed that the total mean measurement time for the IC in water was 29 min and for the ICP was 11 min with standard deviations of 8 and 6 min, respectively. In addition, setup and breakdown also required less time for the ICP than the IC. The estimated overall time for the IC was 66 min and for the ICP was 33 min, a reduction of 57%. Individual timing of monthly dosimetry showed that ICP measurements could be completed within 15 min, including setup and breakdown.

**TABLE 2:** Total time distribution for ICP versus IC measurements for monthly output.

	Ion chamber in water		IC Profiler w/QW	
Task	Photon	Electron	Photon	Electron
Device setup	∼20	∼2	∼5	∼2
Measurement	**15 (8)**	**14 (8)**	**12 (7)**	**13 (11)**
Breakdown	∼15	NA	∼5	NA
Subtotal	50 (8)	16 (8)	25 (10)	8 (3)
Total	66 (11)		33 (10)	

All unites are in minutes (standard deviation). IC, ionization chamber; ICP, IC Profiler; NA, not available; QW, quad wedge.

## 4 DISCUSSION

Safe implementation of the ICP for monthly QA dosimetry requires an understanding of accuracy, reproducibility, and clinical factors that may affect measurements. The purpose of this work is to describe the author's institutional experience in commissioning and benchmarking the ICP into monthly QA dosimetry. The author's institution implemented the ICP with SunCHECK Machine into monthly QA in late 2018, and gradually expanded its use for additional QA tasks. During the initial implementation and subsequent expansion of the ICP in monthly QA, policy and procedures were written and procedural changes were presented in monthly departmental meetings in an effort to standardize clinical use of the ICP. While performing ICP measurements, conventional IC measurements were also completed to safely assess the difference between the ICP and IC measurements to characterize ICP accuracy. The data obtained in this study was retrospectively analyzed in an effort to make the ICP as the primary measurement device for monthly dosimetry QA.

When implementing the ICP with QWs using SunCHECK Machine, there are considerations that affect ICP accuracy, primarily the gain setting. The gain settings in SunCHECK Machine are used for background collection and dose measurements and can be independently set for each energy's dosimetry template. In the current version of the software (Version 3.2.1), the user is not prompted to recollect background when moving to a subsequent dosimetry template with different gain, a feature present in the standalone Profiler software. Therefore, the gain in SunCHECK machine should be set to a single value for all energies and modalities. After correcting ICP gain settings, linear accelerator output measurements using the ICP showed almost perfect match with IC measurements (0.02% deviation), with a standard deviation of 0.4%. The difference between ICP and IC was not compared with TG‐142 tolerances directly, instead ICP data showed no false‐positives or false‐negative detection with respect to needed adjustments above 2% from unity output as recommended by TG‐142. The change in gain setting also improved the ICP measurement of beam profile constancy parameters, specifically symmetry, which showed 1.3% of measurements failed at a tolerance of 1% compared to 0% postadjustment. Gain settings did not impact the measurement of other dosimetric parameters studied in this work.

The high performance of the ICP presented in this study is unique to the measurement system, the ICP and QWs, integrated with SunCHECK Machine and to the measurement of monthly dosimetry QA parameters on linear accelerators. ICP characterization performed in previous studies have provided the groundwork for this work. These works have shown feasibility of the measurement of beam parameters, usually in non‐clinical settings. Notably, Simon et al. performed initial characterization on the stability and reproducibility of the ICP along with any effects from dose, dose rate, pulse rate frequency, energy, backscatter, and calibration loss. In addition, beam profiles were extensively studied, which characterized the accuracy of the field profiles with comparison to an IC. Additionally, Gao et al. and SNC investigated a proof of principle and the implementation of the ICP with QW system for profile and energy measurements. The scope of this study was to expand ICP characterization by specifically focusing on how the ICP responds in clinical measurements setups and for parameters that include beam output, energy, and profile constancy, as well as relative output parameters such as wedge factor, dose rate constancy, and gating constancy.

Although the ICP performance presented in this work is unique to the ICP equipped with QWs and controlled in SunCHECK Machine, Skinner et al. showed that output measurement using the standalone Profiler software was feasible and showed similar accuracy and standard deviation for beam output measurements compared with IC measurements. In addition, the ratio of ICP and Farmer chamber in this work as well as the work presented by Skinner et al. showed similar noise of 0.5%–1.0%, which was dominated by uncertainty contribution of IC chamber measurements. The difference between the Profiler software and SunCHECK Machine with respect to output is that the Profiler uses the central detector to determine output while SunCHECK averages counts across the central three detectors on the y‐axis. Although it is anticipated that the ICP performance is similar using both methods, the results and dependencies presented in this work should not be assumed to translate to implementations using Profiler.

This work showed that the accuracy of the ICP was independent of calibrations and template baselining, though calibrations and template baselining in this study were kept up to date after annual QA and ICP repairs. Routine calibrations and baselining tasks allow any variations in the stability of the chamber to be accounted for by adjusting the output of the ICP to match the IC.

Statistical differences of ICP accuracy between energy and linear accelerator machine were observed in this study for beam output, profile constancy, and energy; however, clinically all energy and machine responses were within one standard deviation of zero. These tests were performed to eliminate any bias that may occur if the ICP response for a specific machine or energy is varied from the overall group and skewing the full dataset. In addition, these tests are specifically important for standardization, and for determining any underperforming measurements, as the author's institution used multiple ICPs at various sites with QA performed by multiple rotating physicists.

AAPM guidelines outlined by TG‐142 and TG‐198 specifically require the use of a calibrated IC for monthly constancy tests. In our institution, all machine output adjustments, postmaintenance, or annual machine QA TG‐51 are performed using an ADCL Farmer‐type IC in a 1D water tank, with these measurements used to correlate to ICP response. In such an implementation, the ICP is effectively cross‐calibrated to the IC whenever an ICP baseline is reset in SunCHECK Machine.

In addition to linear accelerator output, it was found that all dosimetric monthly QA outlined by TG‐142 could be performed using the ICP, including profile constancy, beam energy, secondary dose rate, wedge factors, and gating constancy. Previous studies have shown the feasibility of using the ICP for measuring beam energy, both *D*_10_ and *R*_50_, and this work matched the presented accuracy as beam energy constancy standard deviations were 0.3% of photons and 0.01 cm for electrons. Profile constancy, secondary dose rate constancy, wedge factors, and gating constancy also showed minimal deviations that were well within recommended TG‐142 tolerances of ±1 for profile constancy or ±2% for all other parameters. In this study, we followed previous vendor recommendations for energy measurement using the ICP with QW, wherein each energy is recommended to have their own array calibration generated using said energy. Using a single array calibration from a single energy for measurement of different energies was not tested or explored in this work.

With efforts in the community to shift to more streamlined QA, a major advantage of using the ICP for monthly QA is the simplicity and reduced time in setting up the ICP compared to preparing IC in water measurements. Prior to analysis for beam output, dose rate constancy, wedge factor, and gating constancy, the ICP was used institutionally to measure beam profile constancy during monthly QA. As a result, the setup, measurement, and take down time for the ICP was already integrated into the overall monthly QA procedure time and did not add any additional time. By using the ICP for all QA presented, all IC in water measurement time (setup and measurement) will be eliminated from the overall time required for linac monthly QA. This process reduces electrons to a single measurement and photons to 3−4 measurements depending on machine capabilities. Reduction and simplification of these measurements also reduces the number of modifications to equipment setup and reduces the number of overall measurements making the QA process extremely efficient. The reduction of time required for monthly dosimetry QA by 57% through the use of the ICP follows efforts outlined in TG‐198, which recommends simplifying monthly QA testing.

Measurement using Profiler software is possible, as demonstrated by Skinner et al.; however, the proposal of a scripted approach highlights the difficulty and need for proper selection of measurement conditions and parameters, each of which represents a potential failure mode. The use of software that houses task specific measurement parameters, task specific baseline values, device calibrations, interfaces directly with the device, and performs automated comparison and tolerance checks against reference baselines inherently solves these issues and mitigates the numerous potential failure modes that can exist when each of the properties listed above are manually set. In our experience, implementing the equivalent tests in the stand‐alone software packaged with ICP requires maintenance of numerous calibrations and device configuration files that are saved and set locally, representing potential loss of measurement standardization and potential failure modes if the implementation is not correctly copied or moved to other measurement computers or a computer is unknowingly modified. Lastly, measurements and results securely stored in a database system permitted rapid data aggregation and analysis necessary for this study, avoiding manual curation of 4 years of measurements.

The detected deviations in beam output constancy during the variable gain period of our study were very small but not insignificant, with very few instances of false positives that would have triggered an investigation or intervention. Detecting the deviation, determining the cause, and reviewing the results of the intervention was only possible through analysis of a large set of data taken over time. This dataset was only acquired through the authors’ institution's cautious approach to implementing new technology and techniques. Had the ICP and QW system in SunCHECK Machine been implemented without such a thorough validation, this deviation would have gone undetected. This study highlights a safe approach to introducing new technology and techniques into critically important areas in therapeutic medical physics.

## 5 CONCLUSION

This study provides an in‐depth analysis on the use of ICP with QWs integrated with SunCHECK Machine for TG‐142 dosimetry QA measurements, and the factors that may affect the accuracy when compared with IC measurements in water. This 4‐year review demonstrated that ICP measurements fall within TG‐142 tolerances and specifically show the ICP beam output measurement agreed with conventional and currently used IC in water measurements. The author's institution aims to use the ICP as the primary measurement for beam output on a monthly basis, and supplement these measurements with IC in water for months in which a beam output adjustment must be completed. While it is certainly possible, the authors’ institution has not tested a workflow for using the ICP for output adjustment. This proposed change in workflow aims to decrease QA time while also maintaining accurate measurements that have been optimized.

## AUTHOR CONTRIBUTIONS

All authors have contributed to this work. Sameer Taneja is the corresponding author and David Barbee is the senior author.

## CONFLICT OF INTEREST STATEMENT

Sameer Taneja and David L. Barbee report Sun Nuclear Corporation paid for conference travel to speak. The authors’ institution is a reference site for Sun Nuclear Corporation.
